# Supplementary material for: Effects of environmental conditions on healthcare worker wellbeing and quality of care: A qualitative study in Niger
Source: PLOS Glob Public Health. 2023 Dec 20;3(12):e0002590. doi: 10.1371/journal.pgph.0002590 (PMC10732385; doi:10.1371/journal.pgph.0002590)
Supplement: Attachment — Submitted filename: Wellbeing and QoC - response to reviewers.docx [file pgph.0002590.s002.docx]

We thank the reviewers and editor for their thoughtful consideration of our manuscript. We appreciate their feedback and its contributions to improving this manuscript. Please see point-by-point responses to reviewers below.

# Reviewer 1

| Summary: This is a very important issue in health care environment for patient’s service and management especially poor environmental conditions. I appreciate to authors to convey this essential issue for health sectors. However study is known phenomena in poor environmental conditions had detrimental effects on quality of care in terms of safety, efficiency, timeliness, patient centeredness, and equity.  Major Issues: Type of case study may be mention in the method section. | We thank the reviewers for their thoughtful consideration of our manuscript. We appreciate their feedback and its contributions to improving this manuscript.  We have classified this study as a descriptive case study, per the typology described in Yin (2014). We have revised the text on p. 3, line 67 to specify the type of case study as descriptive:  “We conducted a descriptive case study in Niger using qualitative interviews with healthcare workers and other staff with job duties in the clinical setting (e.g., cleaners, waste handlers)….”  Yin R. (2014). Case study research and applications: Design and methods (6th ed.). SAGE Publications. |
| --- | --- |
| Minor Issues: Data analysis and presentation may place in different way. | We have made several changes to the methods and results section in response to Reviewer 2. Please see the response to Reviewer 2 (below) for a full description of changes and tracked changes in the manuscript. |
| Other Comments: Curious to know both interviews had taken from same person and questionnaire. | We have clarified this on p. 5, lines 112-117:  “At each HCF, we asked the director to identify staff who worked in the following roles: (1) non-clinical roles for administration, supervision, supply chain management, and procurement related to environmental conditions; and (2) clinical roles interacting with patients and or performing other duties in clinical rooms (e.g., cleaning). From this list, we purposively selected and interviewed one person from each category, who completed one of two interview guides tailored to either clinical or non-clinical duties. For this study, we analyzed the subset of all individuals in clinical roles.” |

# Reviewer #2

| The study is much relevant in the context of resource limited countries. It will be open warming i context of securing the health and safety of both health workers, support staffs, the patients and the attendants in the health care settings. There are few suggestions and comments to improve the manuscript though it reflects hard work of the study team.  Authors are strongly requested to include line numbers in the manuscript text before submission in future or after revision of this manuscript. | We thank the reviewers for their thoughtful consideration of our manuscript. We appreciate their feedback and its contributions to improving this manuscript.  We have included line numbers in the revised submission. |
| --- | --- |
| DMA is supported by a grant from the National Institutes of Environmental Health (T32ES007018). Disclose if his funder have any role in decision to publish the work? | We have clarified the role of this funder as follows (p. 18, lines 530-534):  “This research was funded by World Vision through the Water Institute at UNC. World Vision provided support with designing the study sample, arranging travel and field logistics, and providing supervisory support to the data collection consultant. They had no role in the analysis or decision to publish. DMA is supported by a grant from the National Institute of Environmental Health Sciences (NIEHS) (T32ES007018). NIEHS had no role in the study design, data collection, analysis, or decision to publish.” |
| Regarding data availability statement, please adhere to Journal guidelines. It is true that participants identity must not be revealed in any case. But coded data could be made available on request by ensuring the confidentiality of participants identity. | We appreciate the reviewer’s comments on data availability. We will communicate directly with the editor on this issue, per instructions we have received. |
| Please specify if the first line in the introduction section have specific reference i.e. for the components of environmental condition in HCFs. | We have added the following reference to the first line of the introduction:  Adams, John, Bartram, Jamie & Chartier, Yves. (‎2008)‎. Essential environmental health standards for health care / edited by John Adams, Jamie Bartram, Yves Chartier. World Health Organization. |
| Methods well described. But cleaners and support staffs cannot be called healthcare workers. | Cleaners and waste handlers are classified by the World Health Organization as health workers (see page 14, <https://cdn.who.int/media/docs/default-source/health-workforce/dek/classifying-health-workers.pdf?sfvrsn=7b7a472d_3&download=true>)  We found that in Niger cleaners and waste handlers faced the same impacts on wellbeing and quality of care as nurses and doctors. We find the manuscript is more concise and streamlined if we refer to all participants collectively as healthcare workers rather than repeatedly writing out job titles. |
| Please describe in brief the framework from the Institute of Medicine,[13] (-you can include in the annex the detail but give a brief in the main text) | We have provided additional description of the Institute of Medicine framework on p. 4, lines 80-85:  “We examined the impacts of environmental conditions on quality of care using a framework from the Institute of Medicine.[14] The Institute of Medicine framework was initially proposed in a report that identified deficiencies in the quality of healthcare in the United States and identified six dimensions of quality care (safety, patient-centeredness, timeliness, efficiency, equity, and effectiveness), which could be targeted for improvement. These dimensions have since been widely applied in studies evaluating quality of care in the United States and other countries.” |
| Please include appropriate references in study site description. Are the data described obtained from World Vision program or from National data? | These data were collected by the research team during site visits. We have clarified this in the manuscript and provided relevant data as an SI file.  p. 4, lines 99-100:  “As of October 2022, when data collection for this study was completed, surveys conducted by the research team (SI file 1) indicated that 64% of HCFs had an improved water source on site.…” |
| In Sampling please indicate how the sampling was done (i.e. how much HCFs were selected and the number of interviews or observations were conducted). You could also want to include response rate. | We have clarified the HCF sampling strategy on p. 4, lines 88-91:  “This study was conducted in partnership with the non-governmental organization World Vision. We purposively selected all HCFs in which World Vision had planned or executed improvements to environmental conditions as of October 2022, as part of an ongoing infrastructure improvement program.”  We revised the text to provide the response rate on p. 5, lines 118-119:  “All individuals who were approached to participate in the study agreed to participate.”  The number of interviews and demographic data on participants is broken down in detail in Table 1. |
| Results section; Results well formatted and well summarized in the tables but should be backed up by rich verbatim n the descriptions. Please include reliable verbatim on the physical impact on wellbeing. Please include relatable verbatim as well in Mental impact (Stress). The finding will be much relatable and strong by the use of appropriate verbatim and readers can visualize the scenario which is the worth of qualitative studies. | We have added quotes as suggested to the following sections. Please see tracked changes for details.   - Physical wellbeing (chemical) - Mental wellbeing (Stress) - Economic wellbeing (unpaid labor) - Quality of care (patient centeredness) |
| If applicable please include relatable pictures in the reliable sections or include them in the annexes (please adhere to Journal criteria while adding pictures and graphics) | This is not applicable. We performed thematic analysis of transcripts from in-depth interviews, and we did use any qualitative methods that involved collection or analysis of photographs. Pictures of the study site or participants is not the norm in similar qualitative research published in *PLOS Global Health* and risks revealing the specific identities of included healthcare facilities and participants. |
| In the introduction of discussion section; the last line would be The facilities where environmental conditions were improved, participants also reported corresponding improvements in wellbeing and quality of care in most cases. | Thank you. We have corrected this typo. |
| Limitations well explained  Implications well described with strong recommendations for future research. | We thank the reviewers for their feedback. We have left the limitations and implications sections as-is. |
